# Boltzmann- and Non-Boltzmann-Based Thermometers in the First, Second and Third Biological Windows for the SrF_2_:Yb^3+^, Ho^3+^ Nanocrystals Under 980, 940 and 915 nm Excitations

**DOI:** 10.1186/s11671-022-03718-z

**Published:** 2022-08-30

**Authors:** Linxuan Wang, Liang Li, Maohui Yuan, Zining Yang, Kai Han, Hongyan Wang, Xiaojun Xu

**Affiliations:** 1 College of Advanced Interdisciplinary Studies, National University of Defense Technology, Changsha, 410073 China; 2 State Key Laboratory of Pulsed Power Laser Technology, National University of Defense Technology, Changsha, 410073 China; 3 Hunan Provincial Key Laboratory of High Energy Laser Technology, National University of Defense Technology, Changsha, 410073 China

**Keywords:** NIR luminescence, Non-thermally coupled energy levels, Wide range temperature sensing, Tri-wavelength excitations

## Abstract

Spectrally determination of temperature based on the lanthanide-doped nanocrystals (NCs) is a vital strategy to noninvasively measure the temperature in practical applications. Here, we synthesized a series of SrF_2_:Yb^3+^/Ho^3+^ NCs and simultaneously observed the efficient visible upconversion luminescence (UCL) and near-infrared (NIR) downconversion luminescence (DCL) under 980, 940 and 915 nm excitations. Subsequently, these NCs were further utilized for thermometers based on the Boltzmann (thermally coupled levels, TCLs) and non-Boltzmann (non-thermally coupled levels, NTCLs) of Ho^3+^ ions in the first (~ 650 nm), second (~ 1012 nm) and third (~ 2020 nm) biological windows (BW-I, BW-II and BW-III) under tri-wavelength excitations. The thermometric parameters including the relative sensitivity ($S_{{\text{r}}}$) and temperature uncertainty ($\delta T$) are quantitatively determined on the *I*_648_/*I*_541_ (BW-I), *I*_1186_/*I*_1012_ (BW-II), and *I*_1950_/*I*_2020_ (BW-III) transitions of Ho^3+^ ions in the temperature range of 303–573 K. Comparative experimental results demonstrated that the thermometer has superior performances.

**Supplementary Information:**

The online version contains supplementary material available at 10.1186/s11671-022-03718-z.

## Introduction

As a basic physical variable, the temperature is closely related to human life and practical activities. Traditional thermometers possess a relatively large size and need to be physically contacted with the measured object, which severely limits the accuracy of temperature detection and restricts the use of many fields such as biological issues, microelectronic circuits, and nanoscale applications [1]. Therefore, accurate, fast and noninvasive measurement of the temperature is of great significance to practical applications. Lanthanide-doped NCs based on FIR (fluorescence intensity ratio ) or LIR (luminescence intensity ratio) technology could be used as luminescent thermometers which have been extensively developed recently [2–5]. The reason is that lanthanide ions have abundant energy levels and their emissions are heavily dependent on the temperature. These lanthanide-doped luminescent thermometers show the advantages such as fast response, high spatial resolution, high sensitivity, wide adaptability, small error caused by power fluctuation of excitation light source and fluorescence loss [6–10].

Generally, the most present research works have focused on the TCLs of lanthanide ions which the $\Delta E$ is limited to 200–2000 cm^−1^ because this can ensure that the two levels are spectrally separated and not too far apart leading to the variation in thermalization is insignificantly observed [11, 12]. Nevertheless, this additional requirement greatly restrains the utility of numerous NTCLs in lanthanide ions. Thus, the thermometers based on the NTCLs, which are no longer restricted to limitation of $\Delta E$ and can expand the detection wavelengths in a relatively wide range, can achieve relatively high $S_{{\text{r}}}$ and low $\delta T$ and further expand their applications [13–15]. Generally, the quantitative NTCLs model is established by using the Arrhenius equation which can break the restriction of the $\Delta E$ between the NTCLs and well predict the FIR and the accuracy of temperature measurement. As is well known, compared to the strong scattering and absorption effects of visible light in biological tissues, the so-called first (BW-I: 650–950 nm), second (BW-II: 950–1700 nm) and third (BW-III: 1700–2500 nm) optically transparent BWs in the range of 650–2500 nm possess strong tissue penetration and have less scattering, low absorption and weak spontaneous luminescence. Hence, the design of a thermometer, which can simultaneously measure the temperature in the BW-I, BW-II and BW-III, has practical significance and needs in biological applications.

Up till now, many lanthanide ions have been used for temperature measurement, including Yb^3+^ and Ho^3+^ ions [16–19]. On the one hand, Yb^3+^ ions have a large absorption cross section, no excited-state absorption, and a wide absorption spectrum (800–1100 nm) and emission spectrum (975–1200 nm) [20, 21]. On the other hand, Ho^3+^ ions have abundant stepped energy levels and can effectively emit luminescence in a wide range from visible to NIR bands when co-doped with Yb^3+^ ions. Generally, the previously reported Ho^3+^-based thermometers almost utilize the two TCLs of ^5^F_4_ and ^5^S_2_ centered at approximately 540 nm in the visible light based on the Boltzmann theory [22]. In addition, the traditional excitation laser wavelength is 980 nm which could lead to severe heat absorption by the water molecules, thus extremely restraining its application in biological issues. Actually, the Yb^3+^ ions have appreciable absorption capability in other excitation wavelengths (such as 915 nm) where the water absorption coefficient is relatively low. Therefore, exploring the thermometers under different wavelength excitations, especially covering the three biological windows, has very important value in biological applications. However, there still lacks the corresponding research on this aspect [23–25].

In this work, we synthesized a sequence of SrF_2_:Yb^3+^/Ho^3+^ (12/x mol%) NCs doped with different Ho^3+^ concentrations by hydrothermal method. We further investigated the doping Ho^3+^ concentrations dependent on both the UCL and DCL properties, as well as their mechanism of populations and transitions. Subsequently, the SrF_2_:Yb^3+^/Ho^3+^ (12/0.1 mol%) NCs with the stronger luminescence were selected to study the thermal effect under 980, 940 and 915 nm continuous-wave (CW) lasers with the same pumping power density of 110 W cm^−2^. In addition, we innovatively investigated the temperature-dependent luminescence based on TCLs and NTCLs simultaneously under tri-wavelength excitations. The quantitative model we used successfully calculated the FIRs and determined the parameters of the thermometer in the first, second and third biological windows.

## Experimental Sections

### Synthesis of SrF_2_:Yb^3+^/Ho^3+^ NCs

The chemicals of SrCl_2_·6H_2_O (99.99%), YbCl_3_·6H_2_O (99.9%), HoCl_3_·6H_2_O (99.9%), Na_3_C_6_H_5_O_7_ (98%) and NH_4_F (98%) were purchased from Aladdin (China). The synthesis procedure of NCs by a hydrothermal method is similar to our previous literature [26]. Take SrF_2_:Yb^3+^/Ho^3+^ (12/0.1 mol%) NCs as an example. Firstly, 1.758 mmol SrCl_2_, 0.24 mmol YbCl_3_ and 0.002 mmol HoCl_3_ were dissolved in 10 mL deionized water and stirred for 1 h. Secondly, 10 mL Na_3_C_6_H_5_O_7_ (1 M) and 20 mL NH_4_F (1 M) aqueous solutions were added to the above mixed solutions and sequentially stirred for another 1 h. Lastly, the mixtures were transferred into a 50 mL Teflon-lined autoclave and heated at 200 °C for 8 h. When the autoclave was naturally cooled down to room temperature, the as-prepared SrF_2_ NCs were collected by centrifugation at 6000 rpm for 4 min and washed with ethanol and deionized water several times. The final products were dried in an oven at 60 °C for 12 h, and finally, the white powders were obtained for further use.

### Characterization

The morphology and size of the as-prepared SrF_2_ NCs were characterized by transmission electron microscopy (TEM). X-ray diffraction (XRD) patterns were measured using a powder diffractometer (Bruker D8 advance). The DCL and UCL spectra of SrF_2_:Yb^3+^/Ho^3+^ NCs were measured by a fluorescence spectrophotometer (Zolix Omni-l3072i) coupled with an R928 photomultiplier tube (PMT) for visible light detection and an InGaAs avalanche photodetector (ZPS-PN15) for NIR emissions collection. The excitation sources are semiconductor lasers with different wavelengths of 980, 940, and 915 nm. The UV–Vis-NIR spectra of SrF_2_:Yb^3+^/Ho^3+^ (12/0.1 mol%) NCs were recorded in diffuse reflectance mode in the range of 400–2200 nm by using PerkinElmer Lamda-750 UV–Vis-NIR spectrometer unit. For temperature-dependent luminescence measurement, a temperature controller (RT 600, Shanghai Hotz Instrument Technology Co., Ltd) was used to change and control the temperature. At room temperature, the spectra in the wavelength range of 400–2200 nm were collected using the constructed experiment system. Then, the sample was heated by the temperature controller, and the spectra were detected with a step of 25 K in the range of 303–573 K. Especially, we set the heating rate of the temperature controller to 12.5 K min^−1^ and kept the constant temperature for 12 min to ensure that the temperature of sample always reached the scheduled temperature during the spectrum acquisition process.

## Results and Discussion

### Structure Characterization

**Fig. 1:**
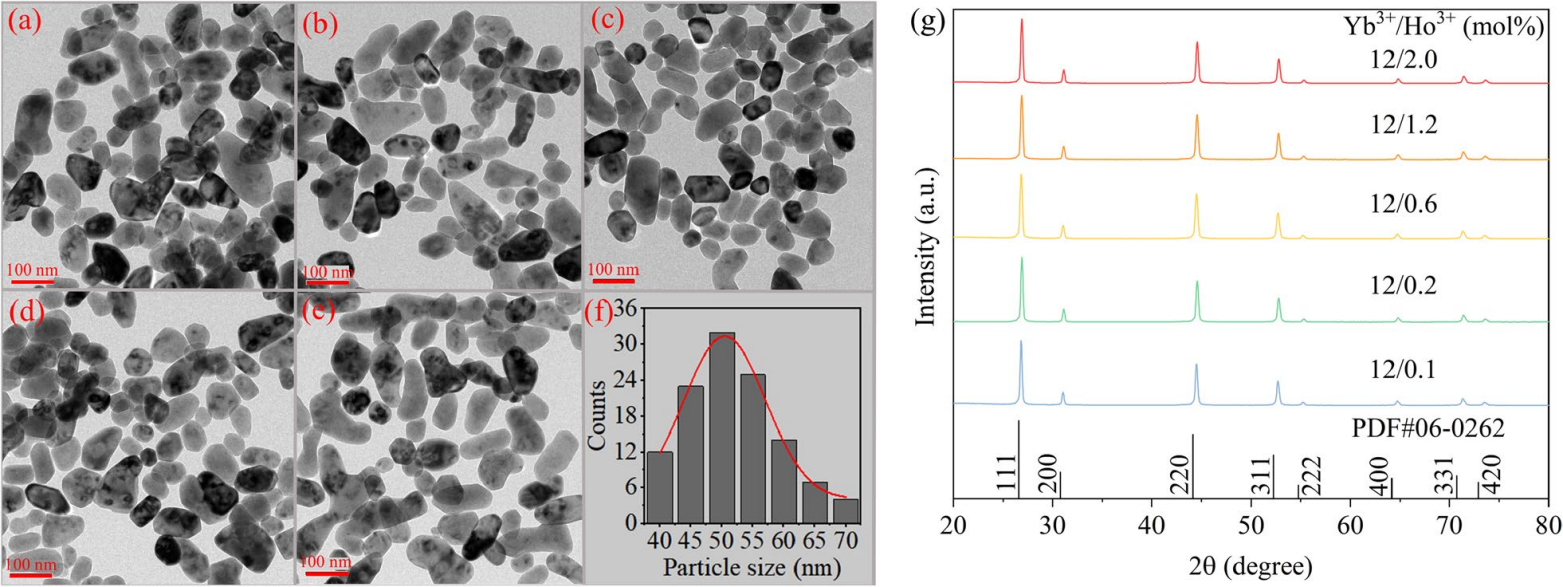
TEM images of SrF_2_:Yb^3+^/Ho^3+^(12/x mol%) NCs, **a** x = 0.1, **b** x = 0.2, **c** x = 0.6, **d** x = 1.2, **e** x = 2.0. **f** The particle size distribution of SrF_2_:Yb^3+^/Ho^3+^ (12/0.1 mol%) NCs. **g** XRD patterns of SrF_2_:Yb^3+^/Ho^3+^ (12/x mol%) NCs doped with different Ho^3+^ concentrations

Figure 1a–e shows the TEM images of SrF_2_:Yb^3+^/Ho^3+^(12/x mol%) doped with different Ho^3+^ concentrations. The morphology of these NCs exhibits an ellipse or rectangle shape. Figure 1f demonstrates the average size of these synthesized NCs is about 50 nm. As shown in Fig. 1g, XRD patterns further prove that the diffraction peaks of the samples match the standard card of the SrF_2_ phase (JCPDS No. 06-0262) well. Both the TEM and XRD characterizations reveal that the doping of small amounts of Yb^3+^ and Ho^3+^ ions has almost no effect on the lattice structure and morphology of the SrF_2_ NCs.

### DCL and UCL Properties

**Fig. 2:**
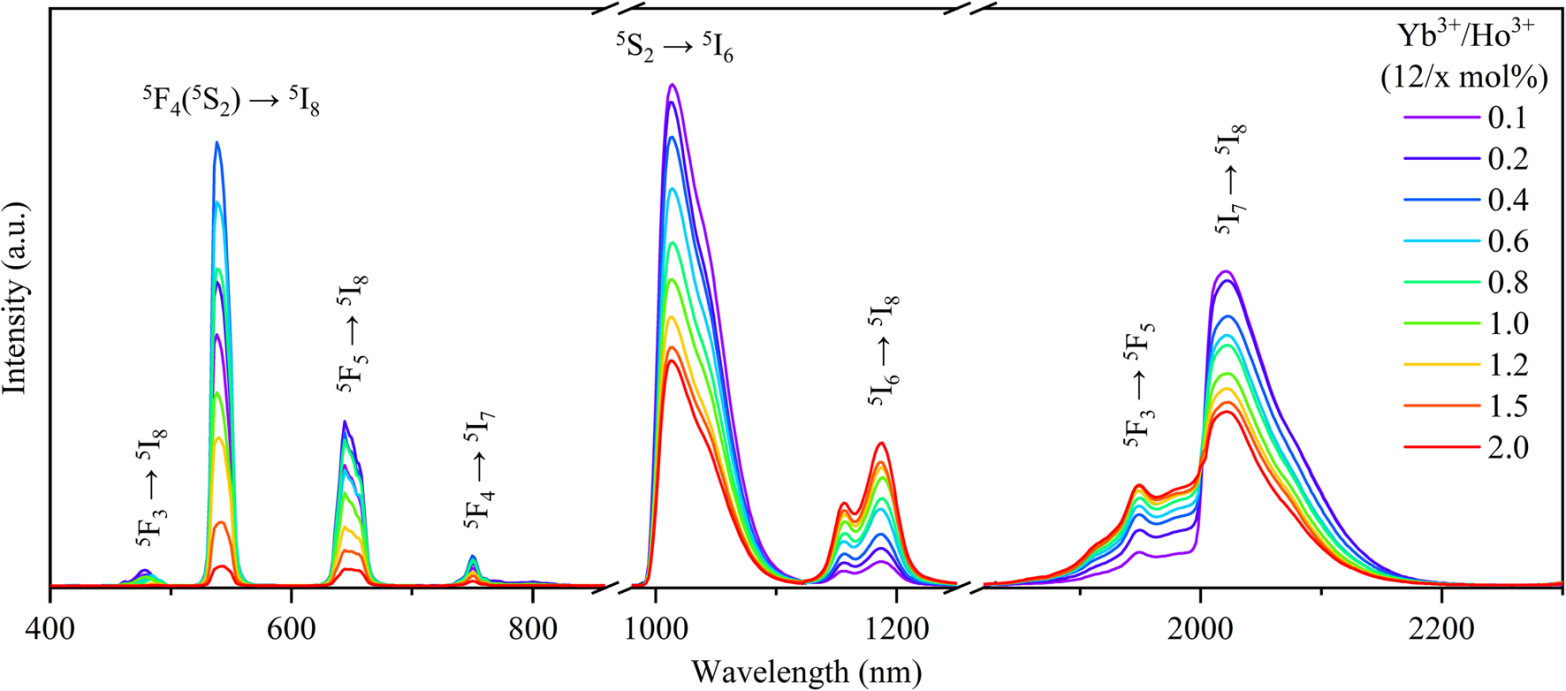
The visible UCL and NIR DCL spectra of SrF_2_:Yb^3+^/Ho^3+^ (12/x mol%) NCs doped with different Ho^3+^ concentrations under the excitation of 980 nm CW laser

**Fig. 3:**
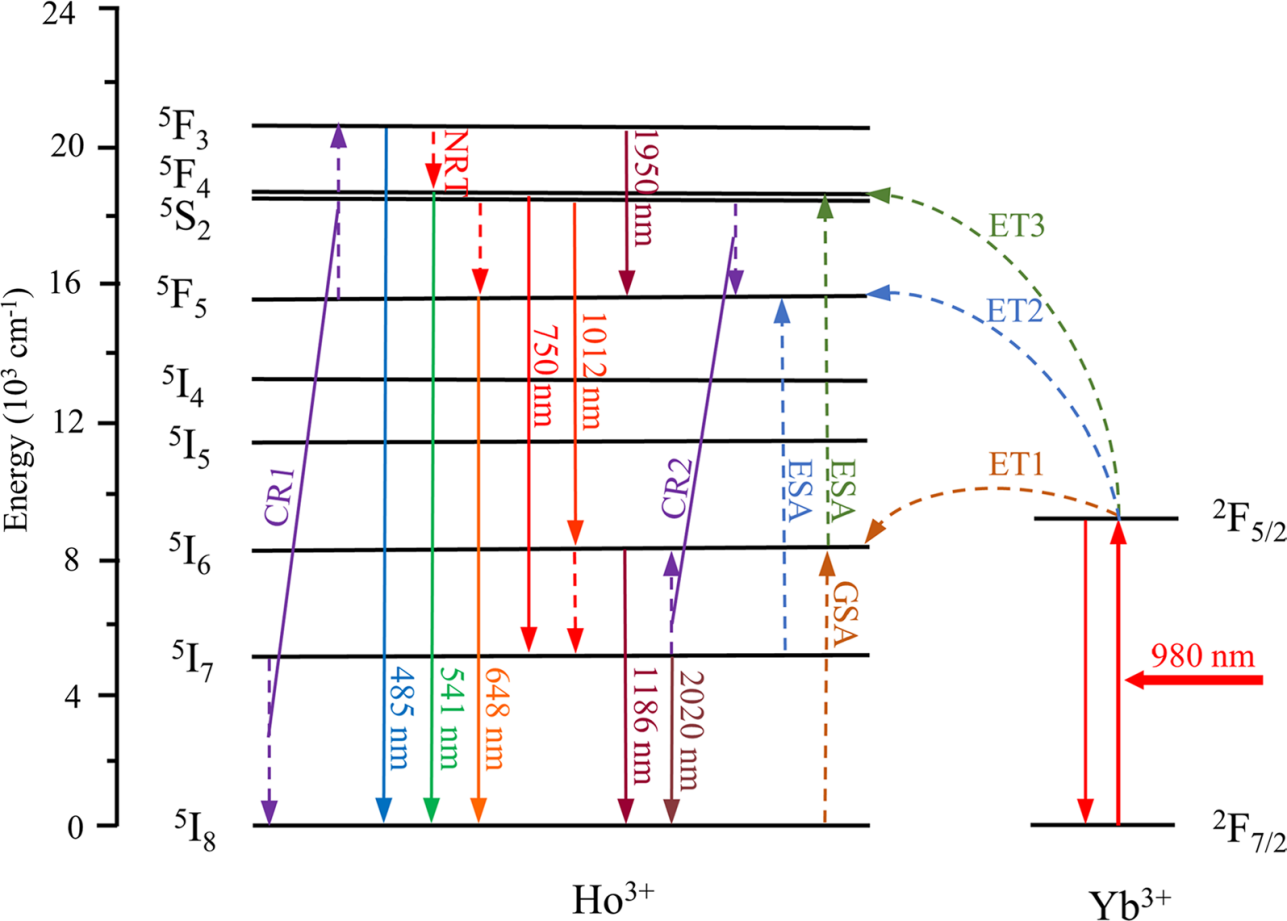
The energy level diagram for SrF_2_:Yb^3+^/Ho^3+^ NCs excited at 980 nm. The corresponding processes of ET, ESA, CR and NRT are also provided

In our preliminary experiment as shown in Additional file 1: Fig. S1, when the doping concentration of Ho^3+^ was fixed at 0.1 mol%, the intensity of UCL firstly increased and then decreased with the increase in Yb^3+^ concentration. The luminescence intensity reached its maximum when the concentration of Yb^3+^ was 12 mol%. Therefore, the Yb^3+^ concentration was fixed at 12 mol% and further investigated the dependence of the luminescence intensity on Ho^3+^ doping concentration of SrF_2_:Yb^3+^/Ho^3+^ (12/x mol%) NCs. Figure 2 shows the visible UCL and NIR DCL spectra of SrF_2_:Yb^3+^/Ho^3+^ (12/x mol%) NCs doped with different Ho^3+^ concentrations under the excitation of 980 nm laser. There are eight typical emission bands centered at 485, 541, 648, 750, 1012, 1186, 1950, and 2020 nm, which are ascribed to the transitions of ^5^F_3_ → ^5^I_8_, ^5^F_4_(^5^S_2_) → ^5^I_8_, ^5^F_5_ → ^5^I_8_, ^5^F_4_ → ^5^I_7_, ^5^S_2_ → ^5^I_6_, ^5^I_6_ → ^5^I_8_, ^5^F_3_ → ^5^F_5_ and ^5^I_7_ → ^5^I_8_ from Ho^3+^ ions, respectively. Obviously, the visible UCL and NIR DCL of SrF_2_:Yb^3+^/Ho^3+^ (12/x mol%) NCs exhibit different varying trends when doped with different Ho^3+^ concentration. The visible UCL increases with the increase in Ho^3+^ concentrations within a low concentration range and reaches the maximum at 0.4 mol% Ho^3+^ and then decreases sharply with the increase in Ho^3+^ ions again. Analysis of this phenomenon proves that concentration quenching plays an important role [27]. The increase in Ho^3+^ concentration promotes an increase in rare-earth-ion pair formation in the SrF_2_ lattice which correspondingly reduces the distance between Ho^3+^ ions compared the Yb^3+^–Ho^3+^ ions, thus facilitating the occurrence of cross-relaxation (CR) between the adjacent Ho^3+^ ions [28, 29]. For the NIR DCL part, the 1012 and 2020 nm emission intensities gradually decrease with the increase in Ho^3+^ concentration, whereas the 1186 and 1950 nm emission intensities are opposite. We speculate that this is mainly due to the CR process of Ho^3+^ ions (CR1 and CR2 in Fig. 3). The CR1 process of ^5^I_7_ + ^5^F_5_ → ^5^I_8_ + ^5^F_3_ can promote the population of ^5^F_3_ state and inhibit the population of ^5^I_7_ state, which will enhance the 1950 nm emission and decrease the 2020 nm emission, respectively. Similarly, the CR2 process of ^5^I_7_ + ^5^S_2_ → ^5^I_6_ + ^5^F_5_ enhances the population of ^5^I_6_ state and simultaneously reduces the population of ^5^S_2_ state, thus strengthening the 1186 nm emission and weakening the 1012 nm emission. Notably, the complex excited-state absorption (ESA) and energy transfer (ET) processes can also contribute to the above observed phenomenon.

Figure 3 illustrates the energy level diagram for Yb^3+^ and Ho^3+^ ions under 980 nm excitation, which also contains the ET, ESA, CR and non-radiative transition (NRT). Generally, Yb^3+^ ions can be populated through the ^2^F_7/2_ → ^2^F_5/2_ transition by directly absorbing 980 nm photon and then transferring the energy to adjacent Ho^3+^ ions through successive ET processes to populate the ^5^I_6_, ^5^F_5_ and ^5^F_4_ states of Ho^3+^ [30, 31]. Moreover, the ^5^F_3_ state is populated by the CR (^5^I_7_ + ^5^F_5_ → ^5^I_8_ + ^5^F_3_) process, followed generating the 485 nm (^5^F_3_ → ^5^I_8_) and 1950 nm (^5^F_3_ → ^5^F_5_) emissions. Subsequently, the electrons in the ^5^F_4_ state will transition to the ^5^I_8_ and ^5^I_7_ states, thereby emitting the 541 nm green light and 750 nm red emission, respectively [32, 33]. Simultaneously, partial electrons in the ^5^F_4_ state will populate to the ^5^S_2_ state by the NRT process, subsequently transitioning to the ^5^I_6_ generating NIR emission at 1012 nm. Similarly, the electrons in the ^5^F_5_ state will transition to the ^5^I_8_ state, thereby emitting the 648 nm emission. Additionally, the transitions from the ^5^I_6_ and ^5^I_7_ states to ^5^I_8_ state produce the 1186 nm and 2020 nm NIR emissions, respectively.

**Fig. 4:**
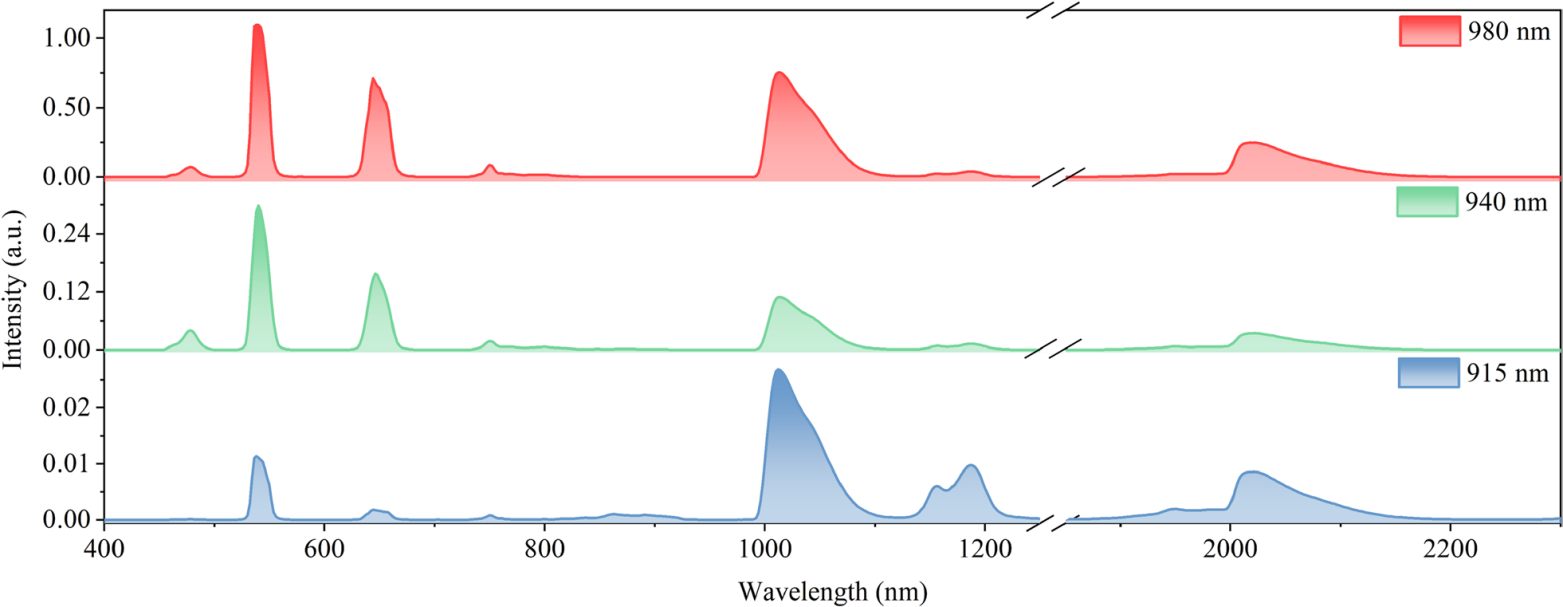
The spectra of SrF_2_:Yb^3+^/Ho^3+^ (12/0.1 mol%) NCs under the 980, 940 and 915 nm excitations, respectively

Notably, except excited by the 980 nm, the Yb^3+^ ions can also be excited at 940 and 915 nm lasers and then transfer energy to Ho^3+^ ions by ET process [34, 35]. To further explore the influence of different excitation sources on the emission spectra for the SrF_2_:Yb^3+^/Ho^3+^ NCs, we further investigate the photoluminescence properties of SrF_2_:Yb^3+^/Ho^3+^ (12/0.1 mol%) NCs under the 980, 940 and 915 nm excitations with the same pumping power density (11 W cm^−2^), as shown in Fig. 4. The results show that emission efficiency under 980 nm excitation is the highest compared with the 915 and 940 nm excitations, indicating that the largest absorption cross section at 980 nm and lowest absorption cross section at 940 and 915 nm. Under the same pumping power density, the intensity of visible emissions under 980 nm excitation is almost 40 times than that under 940 nm excitation and 80 times than 915 nm excitation, while the NIR light is almost 4.5 times than that under 940 nm excitation and nine times than that 915 nm excitation. The quantum yields of SrF_2_: Yb^3+^/Ho^3+^ (12/0.1 mol%) NCs were measured under 980 nm excitation which is ~ 0.51%. Unfortunately, we cannot measure the quantum yield of the SrF_2_:Yb^3+^/Ho^3+^ (12/0.1 mol%) NCs under 940 and 915 nm excitations, which is due to the relatively small absorption cross section and much weak luminescence intensity at these two wavelength excitations than that under 980 nm excitation [36–38]. In addition, the size of synthesized SrF_2_:Yb^3+^/Ho^3+^ NCs is about 50 nm, resulting in a large specific surface area which places the dopant lanthanide ions closer to the surface. This leads to an increase in non-radiative relaxations of the emitting and intermediate levels by solvent molecules.

**Fig. 5:**
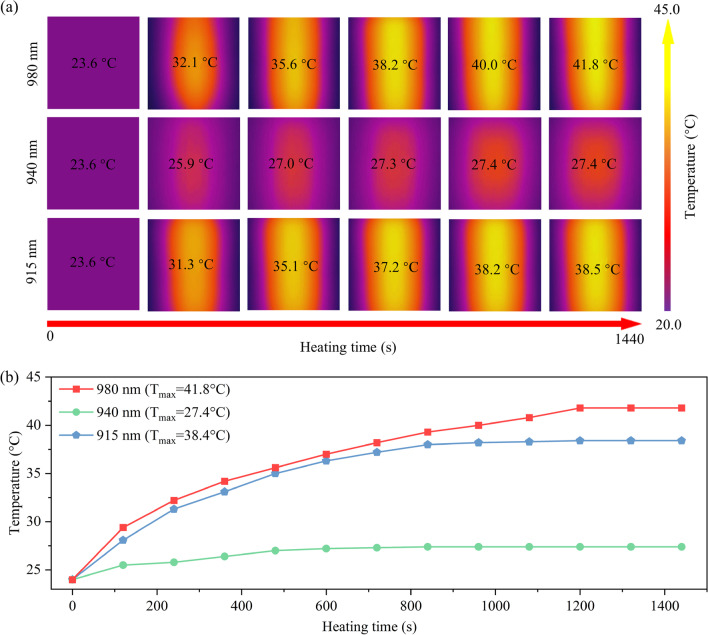
**a** The thermal images and **b** the relationship between temperature and heating time of SrF_2_:Yb^3+^/Ho^3+^ (12/0.1 mol%) NCs under the 980, 940 and 915 nm excitations, respectively

Figure 5a displays the thermal images of SrF_2_:Yb^3+^/Ho^3+^ (12/0.1 mol%) NCs dispersed in ethanol solutions under the 980, 940 and 915 nm laser illustration with a step of 240 s. The excitation power density is 110 W cm^−1^. Over the 1440 s of the testing process, the temperature gradually rises while manifesting different magnitudes for different excitation wavelengths. Figure 5b further depicts the plot of temperature changes as a function of the heating times. During the heating time, the maximum temperature increases up to 38.5 °C and 41.8 °C under 915 and 980 nm excitations, respectively. In contrast, the temperature merely elevates from an initial 23.6 °C to the final 27.4 °C under 940 nm excitation. Obviously, the 940 nm laser-induced heating effect is unconspicuous compared to the 915 and 980 nm lasers. Therefore, the design of a higher sensitivity thermometer which can minimize thermal effects on organisms has more significant significance in the biological and medical fields.

### Ratiometric Temperature Sensing

**Fig. 6:**
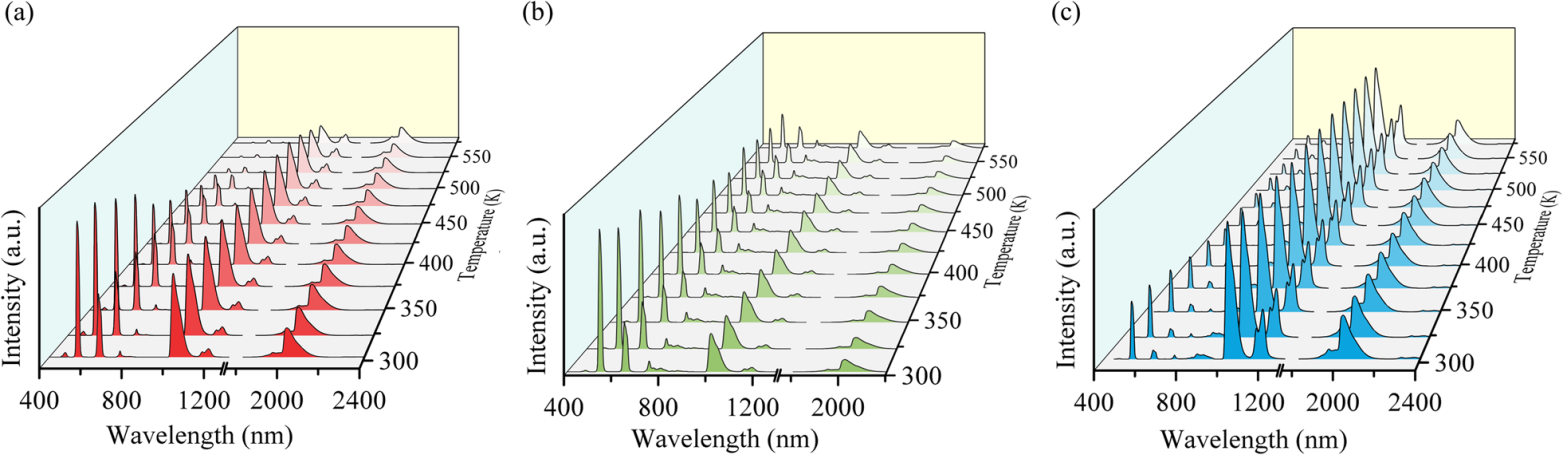
Temperature-dependent spectra of SrF_2_:Yb^3+^/Ho^3+^ (12/0.1 mol%) NCs under the **a** 980 nm, **b** 940 nm and **c** 915 nm excitations, respectively

Having obtained the efficient visible UCL and NIR DCL simultaneously under the excitation of 980, 940 and 915 nm lasers, here, we continue to investigate the ratiometric temperature sensing performances. Figure 6 displays the temperature-dependent spectra of SrF_2_:Yb^3+^/Ho^3+^ (12/0.1 mol%) NCs under the excitation of 980, 940 nm and 915 nm in the range of 303–573 K. Both the visible and NIR emissions are decreasing with the increase in temperature. However, the visible UCL thermally quenches more obviously than the NIR DCL. Under the 980 nm excitation, the intensity of visible 541 nm UCL at room temperature (303 K) is about 95 times than that at the highest temperature of 573 K, while red UCL (648 nm) decreases about 16 times from the 303 to 573 K, as shown in Fig. 6a. On the contrary, the NIR DCL has slight changes when the temperature varies from room temperature to 573 K, which possesses a relative highly thermal stability compared with the visible UCL. Particularly, the NIR DCL remains almost unchanged under the 915 nm excitation.

The temperature-dependent spectra ranging from the BW-I, BW-II and BW-III significantly demonstrate that they can be used for detecting the temperature in a wide range. Considering the actual energy levels of Ho^3+^ ions, especially both TCLs and NTCLs emissions, we choose different methods to analyze and calculate the performances of the Boltzmann-based and non-Boltzmann-based thermometers based on the TCLs or NTCLs. Traditional FIR technology measures the thermal dependence of FIR based on TCLs, which can be defined as follows:

1 $${\text{FIR}}_{B} = \frac{{N_{1} }}{{N_{2} }} = \frac{{I_{1} }}{{I_{2} }} = A\exp \left( { - \frac{\Delta E}{{KT}}} \right)$$

where *N* and *I* represent the populations of the corresponding energy levels and fluorescence intensity, respectively. *A* is the constant that depends on the experimental system, *T* is the absolute temperature and *K* is the Boltzmann constant.

Arrhenius equation is undoubtedly a good method to analyze the mechanism of temperature sensing behavior when using the NTCLs method, which can be expressed as follows [39]:

2 $$I(T) = I_{0} /\left( {1 + B{\text{e}}^{{( - E_{a} /KT)}} } \right)$$

where *I*_0_ is the UCL intensity of the measured NCs at room temperature (*T*_0_), *I*(T) is the UCL intensity at temperature *T*, *B* is the constant and *E*_a_ is the quenching activation energy. The definition of *T* and *K* is the same to Eq. (1).

Although there have been many related studies reported using Arrhenius equation to solve temperature dependence of luminescence intensity due to temperature quenching, in order to further verify the rationality of this equation in dealing with the relationship between Ho^3+^ fluorescence intensity and temperature, we randomly selected the emission intensity centered at 1012 and 2020 nm under 980 nm excitation and obtained the following results through normalization [37, 38]. Additional file 1: Fig. S3a and c shows the dependence of luminescence intensity on the temperature at 1012 nm and 2020 nm, and Additional file 1: Fig. S3b and d displays the fitted results by using Arrhenius equation, respectively. Both the fitting R^2^ values, well-fitted Eq. (2), are greater than 99%. The results indicate that the activation energy is deduced to be 0.27 for 1012 nm and 0.23 eV for 2020 nm, respectively.

Therefore, the FIR based on NTCLs can be modified as follows [40, 41]:

3 $$\begin{aligned}\hbox{FIR}_{N - B} &= \frac{{I_{1} (T)}}{{I_{2} (T)}} = \frac{{I_{0,1} }}{{I_{0,2} }}\frac{{1 + B_{2} \exp ( - E_{2} /\hbox{KT})}}{{1 + B_{1} \exp ( - E_{1} /\hbox{KT})}} \\ & \approx \alpha + \beta \exp \left( - \frac{{\Delta E_{a} }}{\hbox{KT}}\right)\end{aligned}$$

where *I*_1_(T) and *I*_2_(T) represent the UCL intensity of the two corresponding UCL emissions at temperature *T*, respectively. *α* and *β* are constants that are dependent on *I*_0_ and *I*(T). *E*_1_ and *E*_2_ are the corresponding quenching activation energy. $\Delta E_{{\text{a}}}$ is a parameter associated with *E*_1_ and *E*_2_.

**Fig. 7:**
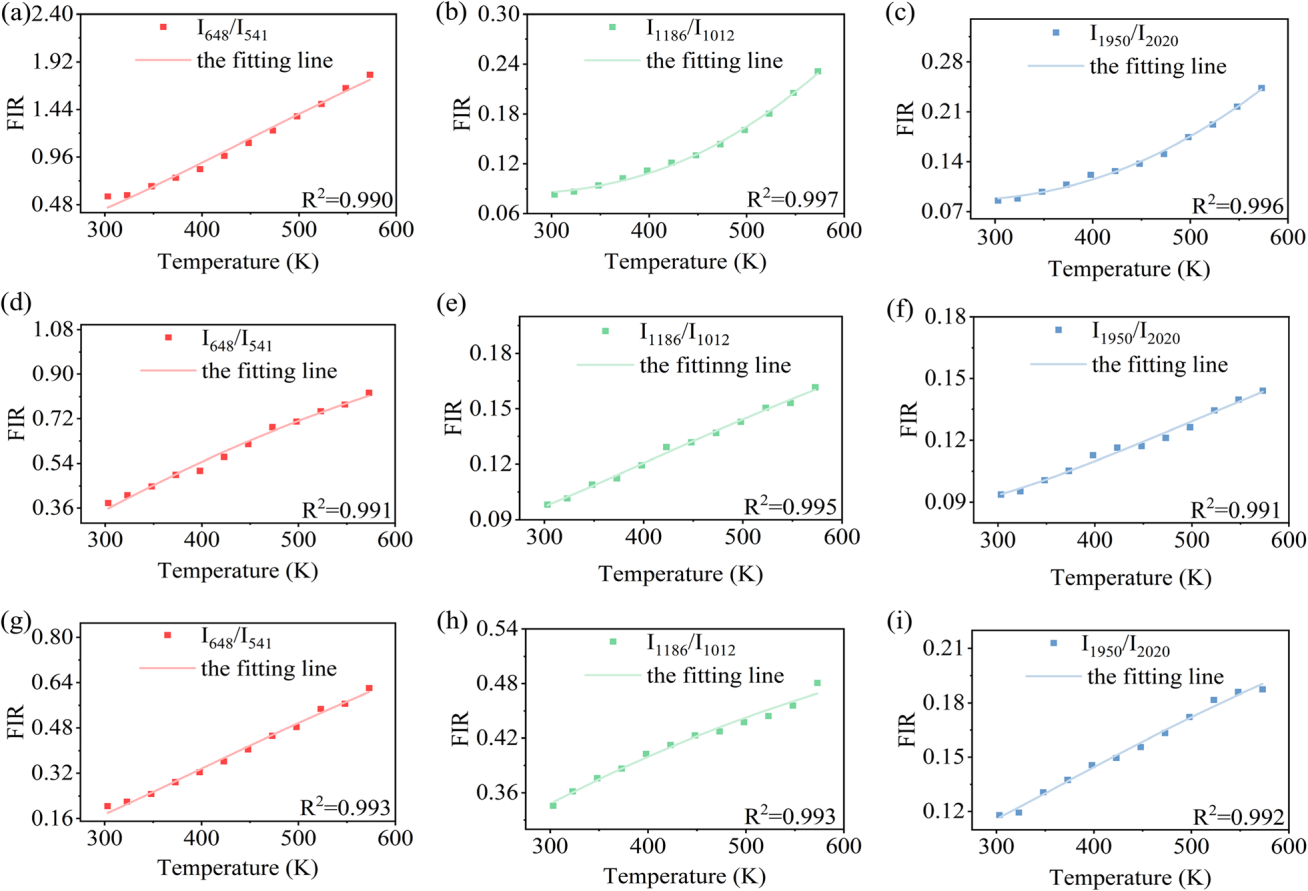
The experimental data (dot) and fitting curves (line) of different FIRs versus temperature of SrF_2_:Yb^3+^/Ho^3+^ (12/0.1 mol%) NCs under the **a**–**c** 980 nm, **d**–**f** 940 nm and **g**–**i** 915 nm excitations, respectively

Figure 7 shows the FIR ratios of *I*_648_/*I*_541_, *I*_1186_/*I*_1012_ and *I*_1950_/*I*_2020_ as a function of the external temperature under tri-wavelength excitations. To ensure the accuracy of experimental data, we fitted the FIR ratios using the Gaussian fitting based on the integrated areas of each UCL peak. As a result, the values of FIR increase with the increase in temperature. Among them, the FIR of *I*_648_/*I*_541_ is fitted with Eq. (1), and the FIR of *I*_1186_/*I*_1012_ and *I*_1950_/*I*_2020_ is fitted with Eq. (3). All the fitting *R*^2^ values of curves are greater than 99.0%, indicating that the rationality of the FIR model is based on the TCLs and NTCLs.

To better evaluate the capability of a thermometer, the ${\text{S}}_{\text{R}}$ is used to represent the relative sensitivity of the thermometer, which is defined as follows [42, 43]:

4 $$S_{R,B} = \frac{1}{{{\text{FIR}}}}\left| {\frac{{\partial {\text{FIR}}}}{\partial T}} \right| = \frac{\Delta E}{{{\text{KT}}^{2} }}$$

5 $$\begin{aligned}S_{R,N - B} &= \frac{1}{{{\text{FIR}}}}\left| {\frac{{\partial {\text{FIR}}}}{\partial T}} \right| \\ &= \frac{{\Delta E_{{\text{a}}} }}{{{\text{KT}}^{2} }}\frac{{\beta \exp ( - \Delta E_{a} /{\text{KT}})}}{{\alpha + \beta \exp ( - \Delta E_{a} /{\text{KT}})}}\end{aligned}$$

Equations (4) and (5) are the expressions $S_{R}$ based on TCLs and NTCLs, respectively.

**Fig. 8:**
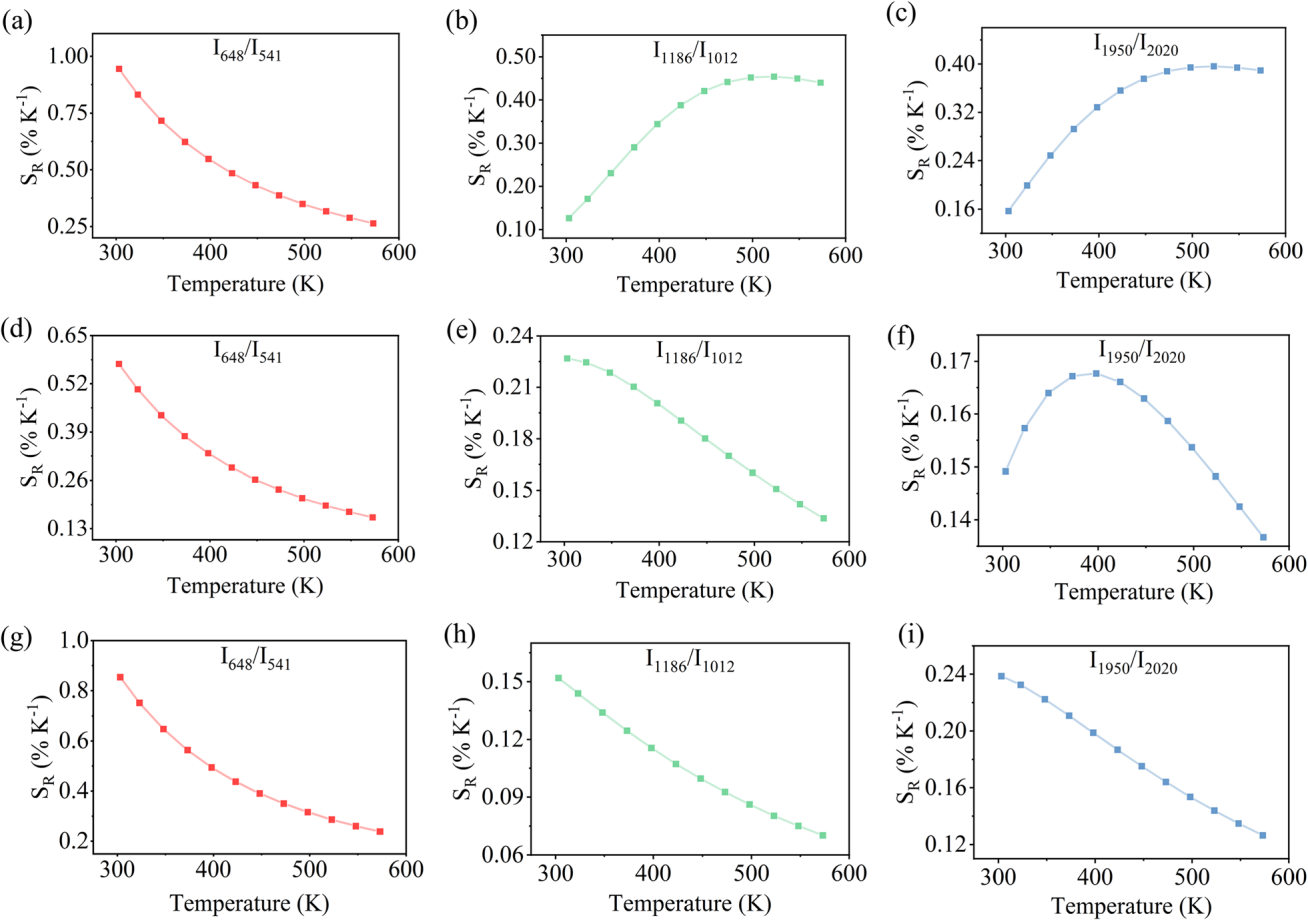
The relative sensitivity ${\text{S}}_{\text{R}}$ versus temperature of SrF_2_:Yb^3+^/Ho^3+^ (12/0.1 mol%) NCs under the excitation of **a**–**c** 980 nm, **d**–**f** 940 nm and **g**–**i** 915 nm, respectively

Figure 8 displays the relative sensitivity of different FIRs based on TCLs and NTCLs dependent on the temperature. In general, for all the UCL emission ratios, the $S_{R}$ under 980 nm excitation is the highest, and the $S_{R}$ under 940 nm excitation is the lowest. Particularly, the maximum $S_{R}$ of *I*_648_/*I*_541_ based on TCLs reaches 0.94% K^−1^, 0.57% K^−1^ and 0.85% K^−1^ at the room temperature of 303 K under tri-wavelength excitations, and the value $S_{R}$ decreases gradually with the increase in temperature which is consistent with that described in Eq. (4). It is interesting to note that the maximum $S_{R}$ of *I*_1186_/*I*_1012_ and *I*_1950_/*I*_2020_ based on NTCLs under 980 nm excitation reaches 0.45% K^−1^ and 0.40% K^−1^ at the same temperature of 523 K. And the maximum $S_{R}$ of *I*_1186_/*I*_1012_ attains 0.23% K^−1^ at 303 K, whereas the maximum $S_{R}$ of *I*_1950_/*I*_2020_ reaches 0.17% K^−1^ at 398 K under 940 nm excitation. This is because the amplitude of fluorescence intensity varies with temperature under different excitation sources discrepantly, as shown in Fig. 6. In particular, the variation in NIR fluorescence intensity under excitation of 940 and 915 nm is significantly smaller than that under excitation of 980 nm, which leads to a higher relative sensitivity under 980 nm excitation.

For comparison, Table 1 summarizes the performances of our determined thermometers and compared them to the previously reported thermometers related to Ho^3+^ ions. The relatively higher performance can be achieved in the range of 303–573 K for FIRs of *I*_648_/*I*_541_, *I*_1186_/*I*_1012_ and *I*_1950_/*I*_2020_ in our experiment compared to the previous Ho^3+^-doped thermometers.

**Table 1:** The parameters of *λ*_ex_, *λ*_em_, maximum $S_{R}$, temperature range of the Ho^3+^-doped materials

Samples	*λ*_ex_ (nm)	*λ*_em_ (nm)	NTCLs or TCLs	Temperature range (K)	$S_{R}$(%∙K^−1^)	References
NaYF_4_: Ho^3+^	447	542, 645	TCLs	113–473	0.93 (302 K)	[12]
BaTiO_3_:Ho^3+^,Yb^3+^	980	539, 547	TCLs	305–515	0.34 (305 K)	[16]
LiYF_4_: Yb^3+^, Ho^3+^	976	655, 546	TCLs	100–500	0.0129 (156 K)	[30]
BiPO_4_:Yb^3+^,Ho^3+^	980	652, 657	TCLs	313–573	0.079 (333 K)	[31]
NBT:Ho^3+^,Yb^3+^	980	546, 757	TCLs	93–300	0.29 (93 K)	[41]
LiYb(WO_4_)_2_:Yb^3+^,Ho^3+^	980	541, 663	TCLs	298–573	0.7 (300 K)	[44]
(K_0.5_Na_0.5_)NbO_3_:Ho^3+^	980	526, 552	TCLs	300–650	0.75 (430 K)	[45]
SrF_2_:Yb^3+^,Ho^3+^	980	648, 541	TCLs	303–573	0.94 (303 K)	This work
		1186, 1012	NTCLs	303–573	0.45 (523 K)	This work
		1950, 2020	NTCLs	303–573	0.40 (523 K)	This work
	940	648, 541	TCLs	303–573	0.57 (303 K)	This work
		1186, 1012	NTCLs	303–573	0.23 (303 K)	This work
		1950, 2020	NTCLs	303–573	0.17 (398 K)	This work
	915	648, 541	TCLs	303–573	0.85 (303 K)	This work
		1186, 1012	NTCLs	303–573	0.15 (303 K)	This work
		1950, 2020	NTCLs	303–573	0.24 (303 K)	This work

In addition to $S_{R}$, the temperature uncertainty of $\delta T$ is a very significant parameter used to evaluate the performance of a thermometer, which is defined as [46]:

6 $$\delta T = \frac{1}{{S_{R} }}\frac{\delta \Delta }{\Delta } \times 100\%$$

where $\Delta$ is the average of measured FIR values in the experiment and $\delta \Delta$ is the uncertainty of the calculated FIR.

**Fig. 9:**
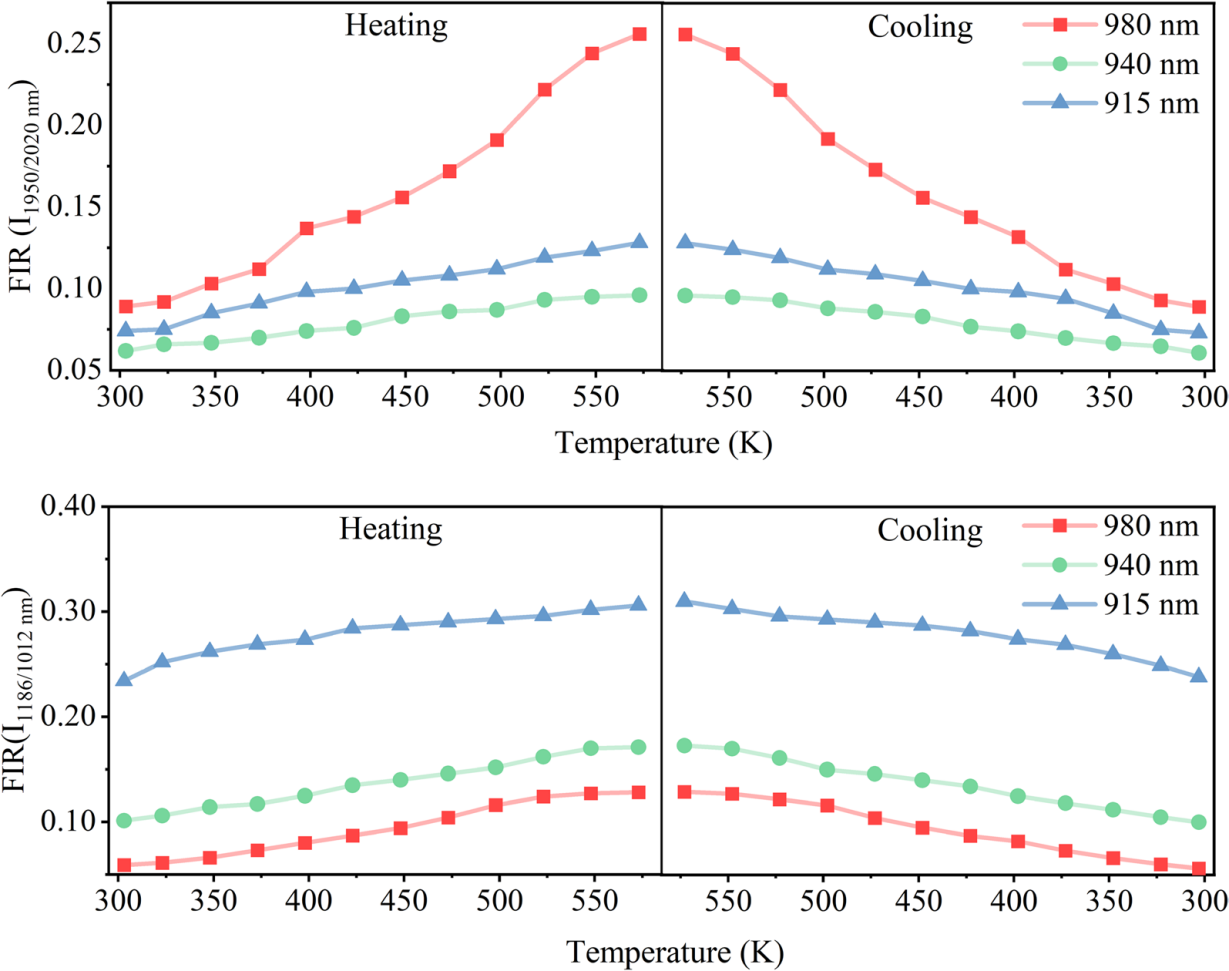
The repeatability of FIR (*I*_1186_/*I*_1012_ and *I*_1950_/*I*_2020_) in SrF_2_:Yb^3+^/Ho^3+^ (12/0.1 mol%) NCs over an arbitrary heating and cooling circle under 980, 940 and 915 nm excitations

Based on Eq. (6), we have calculated the temperature uncertainty of $\delta T$ for the *I*_1950_/*I*_2020_. We have obtained the $\delta T<$ 1.25 K under 980 nm excitation while $\delta T<$ 0.96 K under 915 nm excitation in the temperature range of 303–573 K. In addition, Fig. 9 shows the good repeatability of the temperature-dependent FIR for the NIR bands measured in several heating and colling circles. The results indicate that the thermometer based on NTCLs of Ho^3+^ has relatively high sensitivity and low temperature uncertainty.

## Conclusions

In conclusion, SrF_2_:Yb^3+^/Ho^3+^ (12/x mol%) NCs with an average size of ~ 50 nm were synthesized through the hydrothermal method and characterized by TEM and XRD. Both the efficient NIR DCL and visible UCL are observed under 980, 940 and 915 nm excitations. The doping Ho^3+^ concentrations dependent on the UCL and DCL, as well as their mechanism of population processes and emission transitions, are also discussed. Subsequently, the SrF_2_:Yb^3+^/Ho^3+^ (12/0.1 mol%) NCs exhibited the most intense NIR DCL and visible UCL. Then, these NCs were selected to achieve the Boltzmann- and non-Boltzmann-based thermometers under 980, 940 and 915 nm excitations. The obtained maximum ${\text{S}}_{\text{R}}$ of *I*_648_/*I*_541_ based on TCLs is 0.94% K^−1^ at 303 K, as well as the 0.45% K^−1^ for *I*_1186_/*I*_1012_ and 0.40% K^−1^ for *I*_1950_/*I*_2020_ at 523 K based on NTCLs under 980 nm excitation. The results reveal that these NCs can be applied in biological issues to measure the temperature under different laser wavelength excitations and wide emission bands centered in the first, second and third biological windows.

## Supplementary Information

**Additional file 1: Fig. S1.** Variation of upconversion emission intensity as a function of Yb^3+^ dopant concentration when fixed the concentration of Ho^3+^ (0.1 mol%). **Fig. S2.** The UV–vis–NIR absorption spectra of SrF_2_:Yb^3+^/Ho^3+^ (12/0.1 mol%) NCs. **Fig. S3.** The dependence of luminescence intensity at (a) 1012 nm and (c) 2020 nm of SrF_2_:Yb^3+^/Ho^3+^ NCs on the temperature under 980 nm excitation. Arrhenius equation is used to fit the luminescence intensity dependent on temperature at (b) 1012 nm and (d) 2020 nm.

## Data Availability

The datasets used and/or analyzed during the current study are available from the corresponding author on reasonable request.

## Abbreviations

NCs: Nanocrystals
UCL: Upconversion luminescence
DCL: Downconversion luminescence
NIR: Near-infrared
TCLs: Thermally coupled levels
NTCLs: Non-thermally coupled levels
BW-I: First biological windows
BW-II: Second biological windows
BW-III: Third biological windows
CW: Continuous-wave
TEM: Transmission electron microscopy
XRD: X-ray diffraction
PMT: Photomultiplier tube
CR: Cross-relaxation
ESA: Excited-state absorption
ET: Energy transfer
NRT: Non-radiative transition
