# Supplementary material for: Boltzmann- and Non-Boltzmann-Based Thermometers in the First, Second and Third Biological Windows for the SrF2:Yb3+, Ho3+ Nanocrystals Under 980, 940 and 915 nm Excitations
Source: Nanoscale Res Lett. 2022 Aug 30;17:80. doi: 10.1186/s11671-022-03718-z (PMC9428101; doi:10.1186/s11671-022-03718-z)
Supplement: Supplementary file 1 — Additional file 1: Fig. S1. Variation of upconversion emission intensity as a function of Yb3+ dopant concentration when fixed the concentration of Ho3+ (0.1 mol%). Fig. S2. The UV–vis–NIR absorption spectra of SrF2:Yb3+/Ho3+ (12/0.1 mol%) NCs. Fig. S3. The dependence of luminescence intensity at (a) 1012 nm and (c) 2020 nm of SrF2:Yb3+/Ho3+ NCs on the temperature under 980 nm excitation. Arrhenius equation is used to fit the luminescence intensity dependent on temperature at (b) 1012 nm and (d) 2020 nm. [file 11671_2022_3718_MOESM1_ESM.doc]

**Supplementary material**

Boltzmann- and non-Boltzmann-based thermometers in the first, second and third biological windows for the SrF_2_:Yb^3+^,Ho^3+^ nanocrystals under 980, 940 and 915 nm excitations

Linxuan Wang,^1,2^ Liang Li^,1,2^ Maohui Yuan,^1,2,*^ Zining Yang,^1,2,3^ Kai Han,^1,2^ Hongyan Wang,^1,2,3,**^ and Xiaojun Xu^1,2,3^

*^1^College of Advanced Interdisciplinary Studies, National University of Defense Technology, Changsha, 410073, China*

*^2^State Key Laboratory of Pulsed Power Laser Technology, National University of Defense Technology, Changsha, 410073, China*

*^3^Hunan Provincial Key Laboratory of High Energy Laser Technology, National University of Defense Technology, Changsha, 410073, China*

**yuanmaohuino1@126.com*

[***wanghongyan@nudt.edu.cn*](mailto:**wanghongyan@nudt.edu.cn)

**Fig. S1.** Variation of upconversion emission intensity as a function of Yb^3+^ dopant concentration when fixed the concentration of Ho^3+^ (0.1 mol%).


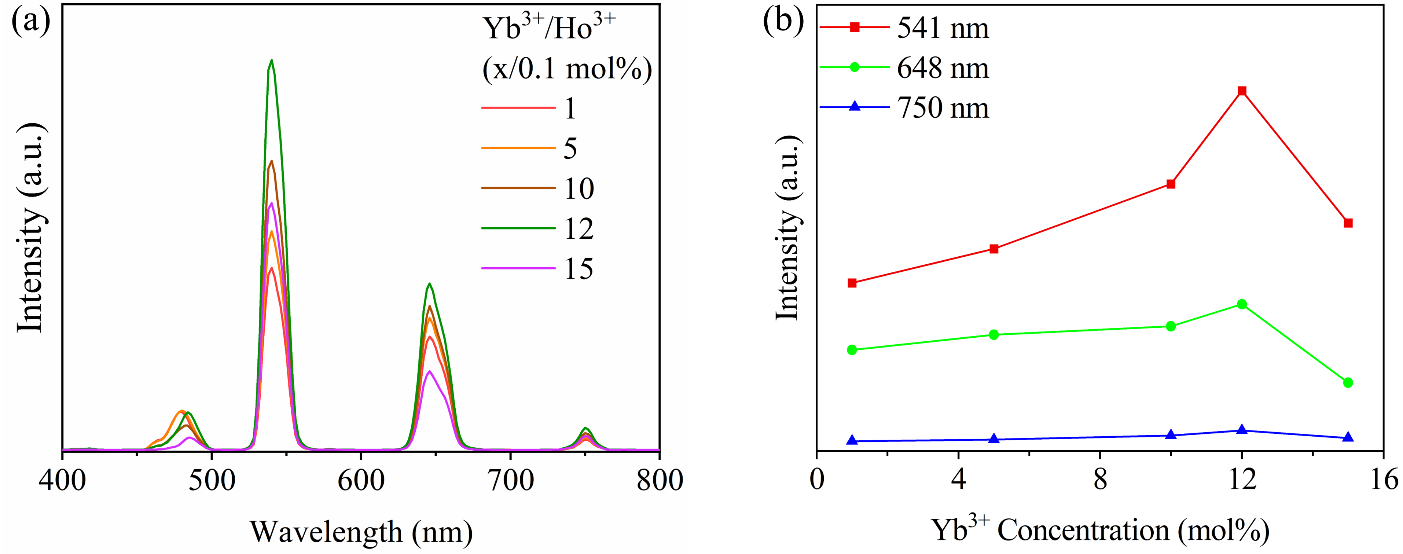


*Part 1*


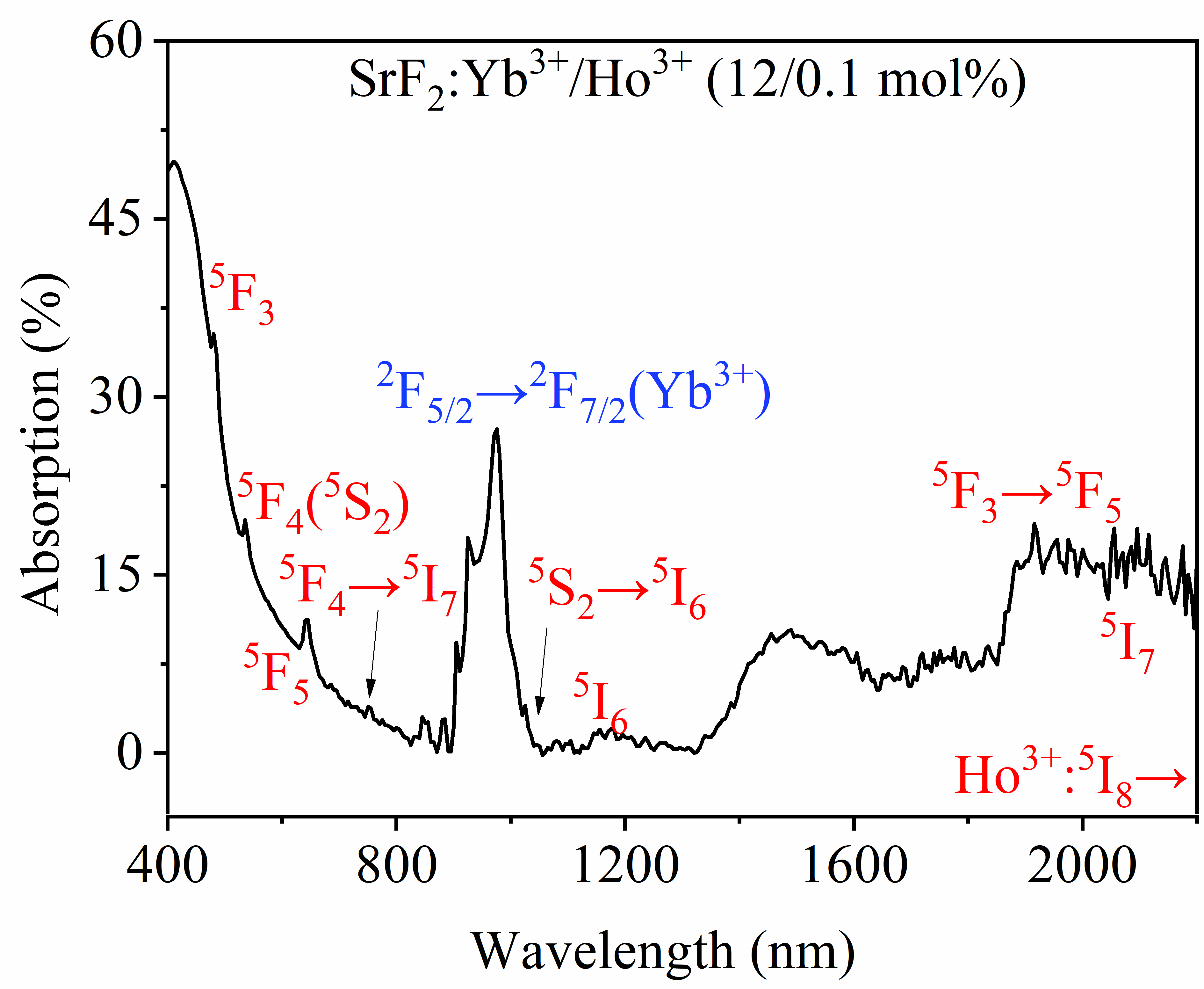


**Fig. S2.** The UV–vis–NIR absorption spectra of SrF_2_:Yb^3+^/Ho^3+^ (12/0.1 mol%) NCs


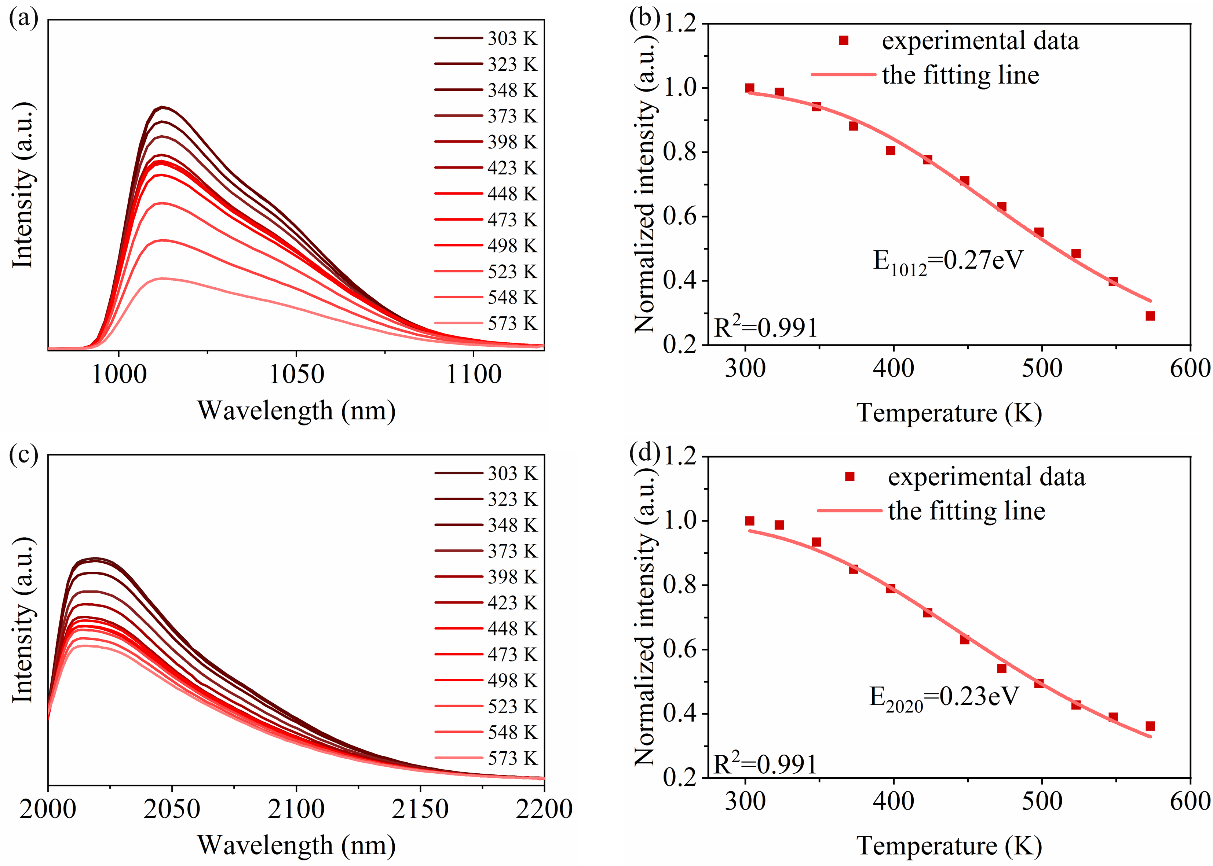


**Fig. S3.** The dependence of luminescence intensity at (a) 1012 nm and (c) 2020 nm of SrF_2_:Yb^3+^/Ho^3+^ NCs on the temperature under 980 nm excitation. Arrhenius equation is used to fit the luminescence intensity dependent on temperature at (b) 1012 nm and (d) 2020 nm.

The formula deduction of Arrhenius equation process is described by using mathematical equivalent changes as follows:

$$\begin{matrix} FIR_{N-B}=\frac{I_{1}(T)}{I_{2}(T)} \\ \text{ }=\frac{I_{0,1}}{I_{0,2}}\frac{1+B_{2}\exp(-E_{2}/KT)}{1+B_{1}\exp(-E_{1}/KT)} \\ \text{ }=\frac{I_{0,1}}{I_{0,2}}\frac{1+B_{1}\exp(-E_{1}/KT)+\left[B_{2}\exp(-E_{2}/KT)-B_{1}\exp(-E_{1}/KT)\right]}{1+B_{1}\exp(-E_{1}/KT)} \\ \text{ }=\frac{I_{0,1}}{I_{0,2}}\left[1+\frac{B_{1}\exp(-E_{1}/KT)(\frac{B_{2}}{B_{1}}\exp((E_{1}-E_{2})/KT)-1)}{1+B_{1}\exp(-E_{1}/KT)}\right] \\ \text{ }=\frac{I_{0,1}}{I_{0,2}}+\frac{I_{0,1}}{I_{0,2}}\frac{B_{1}\exp(-E_{1}/KT)(\frac{B_{2}}{B_{1}}\exp((-\Delta E_{a})/KT)-1)}{1+B_{1}\exp(-E_{1}/KT)} \\ \text{ }\approx\alpha+\beta\exp(-\frac{\Delta E_{a}}{KT}) \end{matrix}$$

Where α, β, and Δ*E*_a_ are parameters associated with the *I*_0_, *B*, and *E*. Specifically, α is approximately related to *I*_0,1_/*I*_0,2_, β is approximately related to *B*_2_/*B*_1_ and *I*_0,1_/*I*_0,2_.
